# Clinical presentation of coronary arteriovenous fistula according to age and anatomic orientation

**Published:** 2015

**Authors:** Hassan Zamani, Mahmoud Meragi, Mohamad Yousef Arabi Moghadam, Behzad Alizadeh, Kazem Babazadeh, Farzad Mokhtari-Esbuie

**Affiliations:** 1 Non-Communicable Pediatric Diseases Research Center, Babol University of Medical Sciences, Babol, IR Iran.; 2 Pediatric Cardiology Department of Shahid Rajaie Heart Center, Iran University of Medical Sciences, Tehran, Iran.; 3 Mashhad University of Medical Sciences, Mashhad. Iran.; 4 Mazandaran university of medical sciences, Sari, Iran.

**Keywords:** Congenital heart disease, Coronary arteriovenous fistula, CAVF.

## Abstract

***Background: ***Coronary arteriovenous fistulas (CAVFs) are direct connections from one or more coronary arteries to cardiac chambers or a large vessel. They are mostly of congenital origin. The aim of this study was to describe clinical presentation and also delineate the course and management of CAVF.

***Methods:*** Clinical data, chest x-rays, echocardiographic and angiographic evaluation of 40 patients with congenital CAVF during 1990 to 2008 were reviewed retrospectively.

***Results:*** Seventeen patients were ≤ 20 years old (42.5%) were mostly asymptomatic, and twenty tree cases were older than 20 years old (57.5%), mostly symptomatic (P<0.05). Twenty one (52.5%) patients had pure CAVF and nineteen (47.5%) patients with associated intarcardiac congenital heart disease (15%) or acquired valvular and coronary arteries diseases (32.5%). CAVFs mostly originated from left anterior descending artery (LAD) (42.5%) and mostly drained into the main pulmonary artery (MPA) (35 %). Twenty-four patients underwent CAVF surgical ligation. From twenty-one patients with pure CAVF, eight (38%) patients were complicated by congestive heart failure and aneurism formation of fistula.

***Conclusion: ***Unlike some previous reports, in our study, the most prevalent origin site for CAVFs was the left anterior descending (LAD). Most patients with CAVFs especially those who went first diagnosed before 20 years old were asymptomatic. On the other hand, as the continuous murmur is not always detected in children or infants, consequently, cases of spontaneous closure may remain undetected. All symptomatic and asymptomatic patients with moderate to severe shunting should be operated on and minimal morbidity and good surgical results could be expected.

Coronary arteriovenous fistulas (CAVFs) are direct connections from one or more coronary arteries to cardiac chambers or a large vessel (1). They are mostly congenital, but rarely may be acquired due to trauma, postcardiac surgery or angioplasty, or very rarely, because of coronary aneurysm rupture (2, 3). CAVFs are present in 0.002% of the general population and are visualized in nearly 0.25% of patients undergoing catheterization (4, 5). Angiographic studies have revealed that the incidence of CAVF ranges from 0.3 to 0.8% (6). Although some previous studies reported CAVFs originate mostly from the right coronary artery (RCA) in approximately 50% of cases (7, 8), some authors mentioned that most CAVFs originate from left arteria descending artery (LAD) (9, 10).

There are, however, certain predilections. More than 90% of fistulae open into right heart chambers or their connecting vessels. True AV fistulae to the veins themselves (Coronary sinus or its major branches or venae cava) are uncommon. Thus, about 40% connect to the right ventricle, 25% to right atrium, 15% to 20% to pulmonary artery, 7% to coronary sinus, and only 1% to superior vena cava (11). Fistulas to the LV are very rare, with an incidence of only 3% (12, 13). 

Unless very large fistula, CAVFs are usually asymptomatic in younger patients. But with increasing age, symptoms begin to appear, and the incidence of complication rises; some people may experience the following symptoms: fatigue, dyspnea, palpitations and ischemic chest pain (14). Heart failure is the most common complication (15). CAVFs may also be incidentally found during diagnostic coronary angiography (16, 17). In this issue, we present our experience in 40 pediatric, adolescent and adult patients with CAVF. The aim of this study was to evaluate the epidemiological aspects and its clinical presentation and management strategies. 

## Methods

We retrospectively reviewed the clinical data of the 40 patients who presented to our institution (Pediatric Cardiology Department of Rajaee Heart Center - Tehran –Iran) with the diagnosis of a congenital CAVF during 1990 to 2008. The acquired type was not entered in the study. The patients were classically categorized in two groups, group1: Pediatric and/or adolescent group (≤20 years of age) and group 2: Adult group (>20 years old). Chest x-ray, echocardiography and angiography were performed for all the patients. The entry site of each fistula was checked and the amount of left to right shunt was calculated. Other concomitant cardiac abnormalities were also noted. The mean follow-up period of the patients was 4.2±4 years. Chi square test and Fisher’s exact test were used to compare the variables and data analysis. Results were considered statistically significant when the variability level was < 0.05. Statistical analysis was performed with SPSS software (Version 15).

## Results

Forty patients previously diagnosed with CAVF were reviewed. The mean age of the patients was 34.9±26.9 years old (between 40 days to 75 years old). Sixteen patients were females (40%), and 24 patients were males (60%) (p<0.05). Seventeen patients were ≤20 years old (group1) (42.5%), and 23 patients were older than 20 years old (group 2) (57.5%) (p<0.05). Transthoracic echocardiography was able to detect CAVF in 14 patients (13 patients in group1 (≤20 years old), and 1 patient in group 2 (>20 years old) (p<0.05).

In group 1, 75% of the patients were asymptomatic (who were incidentally diagnosed in routine physical examination or echocardiography), but only 22.2% of patients in adult group were asymptomatic (p<0.05). Continuous cardiac murmur was the most common sign of the patients with pure CAVF (90.5%), which was detected in all of the patients in ≤20 years old group and 77% of patients who were over 20 years old of age (table1). Twenty-one patients (52.5%) had pure CAVF and the rest had intra-cardiac congenital heart defects (15%) or acquired valvular and coronary artery diseases (32.5%). Associated intracardiac heart defects (CHD) in 6 patients were: Patent ductus arteriosus (PDA) (one patient), pulmonary atresia with intact ventricular septum (one patient), PDA + ventricular septal defect (VSD) (one patient), aorta stenosis (AS) with bicuspid aorta valve (BAV) (2 patients) and atrial septal defect (ASD) +VSD+PDA (one patient). Aneurysm formation of CAVF was seen in 11 patients (27.5%) on admission (4 patients were ≤20 years old and 7 patients in adult group). 

**Table1:** clinical presentation of the patients (21patients)

**variable**	**n**
Continous murmur	19
asymptomatic	11
cardiomegaly	10
exertion dyspenea	5
chest pain	5
fatigue	8
palpitation	2
angina	0

Twenty-four patients went under surgical repair of CAVF, and also the surgical repair of associated heart defects was performed in 10 patients of those patients. No surgical mortality or morbidity was reported. The mean pulmonary to systemic flow ratio (QP/QS ratio) was assessed according to the data, derived from catheterization in 17 patients with no intracardiac congenital heart defects. Surgical repair was performed for all 8 patients with QP/QS≥1.5:1 and also performed for 5 patients with QP/QS<1.5:1 simultaneous with CABG or valve replacement surgery. Another 4 patients with QP/QS<1.5:1 were clinically followed. Cardiomegaly in CXR was significantly detected more common in patients with QP/QS ≥1.5:1 (p<0.05). In our series, the most original site of fistula was the left coronary artery (LCA) and the most common site of drainage was main pulmonary artery (MPA) (35 %) (table 2). Seventy five percent of CAVFs originating from LAD were drained into MPA. Fifty percent of those CAVFs originating from RCA were drained into RV and 50% of CAVFs originating from LMCA were drained into RA (p<0.05). 

**Table 2:** Anatomic Features of CAVFs

Anatomic Features	Group1 <20years old	Group2 >20 years old	total
**Origin of Fistula**			
*Left sided coronary*	3	14	17 (42.5%)
Left Anterior Descending (LAD)	4	3	7 (17.5%)
Left Circumflex (LCX)	5	1	6 (15%)
Left Main Coronary Artery (LMCA)			
*Right Sided Coronary*	5	5	10 (25%)
Right Coronary Artery (RCA)			
**Drainage to**			
*Right-sided chambers*	1+1	13+0	**37 (92.5%)**
Main pulmonary artery + RPA	9+0	1+1	14+1 (35%+2.5%)
Right ventricle + RVOT	4	4	10+1 (25%+2.5%)
Right atrium	3	0	8 (20%)
Coronary sinus	0	2	3 (7.5%)
*Left-sided chambers*	0	1	**3** ** (** **7.5%** **)**
Left ventricle			2 (5%)
Left atrium			1 (2.5%)

RPA: Right pulmonary artery, RVOT: Right ventricle outlet tract.

## Discussion

In this paper, we retrospectively reviewed a clinical data of forty patients with congenital coronary arteriovenous fistula to evaluate the epidemiological aspect and its clinical presentation and management strategies. Although some previous studies have reported CAVFs origin from RCA in approximately 50% of cases (7, 8), in our study, most of CAVFs originated from LAD (42.5%), and only 25% of CAVFs originated from RCA. This result has been compatible with other recently published issues by Tirilomis T (9) and Yusuf Ata (10).

In contrast to the adults, most of the children and adolescent patients with CAVFs are asymptomatic and they only had continuous murmur in routine examination (18-20). In the review study by Liberthson et al., 63% of the patients older than or equal to 20 years old had preoperative symptoms or complications, but only 19% of those less than 20 years of age had preoperative CAVF-related symptoms or complications (20). As a previous result, in our study, 25% of the patients in group 1 (≤20 years old) were symptomatic, but 77.8% of patients in group 2 (>20 years old) had symptoms. Continuous cardiac murmur was the most common sign of the patients with pure CAVF (90.5%), which was detected in all of the patients in group 1 (≤20 years old) and 77% of patients in group 2 (>20 Years old). CAVF-related complications included; Congestive heart failure (CHF) and aneurism formation of fistula were more commonly detected in patients older than 20 years old. There was not any myocardial ischemia or infarction in our series. 

Transthoracic echocardiography can display a dilated coronary artery, where the abnormality arises, and even the fistula itself, including the entrance to a chamber or vessel, in addition to a continuous turbulent systolic and diastolic flow (21). Echocardiography is a primary diagnostic modality in most patients under 20 years of age, but not for the patients older than 20 years old. In our study transthoracic echocardiography was able to detect CAVF in 14 patients (13 patients ≤20 years old, and only 1 patient> 20 years old) (p<0.05). These results probably were due to better echocardiographic windows in this age group. However, coronary angiography is the method of choice for the diagnosis. 

With increasing case reports of spontaneous closure of even large and symptomatic fistulae, the management of the CAVFs especially in asymptomatic children is unclear (22-24) and its dependent on the presence of symptoms, the clinical significance of the fistula, the hemodynamic shunt dimension and the morphological appearance and characteristics of the fistula visualized with different imaging techniques (25). Most authors recommend CAVFs to be closed when there is a significant shunt flow (QP/QS≥1.5:1), or /and when the patient is symptomatic (9, 26, 27), but surgical intervention for asymptomatic patients with little shunt flow is controversy. On the other hand, some investigators prefer operation even in those with no symptoms to prevent fistula related complications that will increase with age, especially because of the risk of heart failure, endocarditis and myocardial ischemia (9, 10, 28). In our study, all patients with shunt flow of QP/QS≥ 1.5: 1 underwent CAVF surgical closure, but the patients with mild shunt flow (QP/QS< 1.5:1) underwent surgical closure of fistula only when these patients had to be operated due to other heart defects including; valves repair or replacement, CABG or other intracardiac congenital heart defects (half of the patients with mild shunt flow). The other half of patients with QP/QS< 1.5: 1 followed-up medically. Surgical related complications are reported 0%-4% among different studies (20). But in our study, there was no surgical morbidity or mortality after surgical repair of fistulas. 

In our study, most of CAVFs with LAD origin were first diagnosed after adulthood (p<0.05), Also, nearly a total of CAVFs drained into MPA were first diagnosed after adulthood (p<0.05). Seventy-five percent of CAVFs originating from LAD in this study were drained into MPA.

The prevalence of CAVFs originated from LAD is likely more frequent than those diagnosed often 20 years of age, since a number of cases drained into to MPA closed spontaneously before this age. 

In this review, most of CAVFs originate from LAD (42.5%), and only 25% of CAVFs originate from RCA, although LAD origin may be underestimated. Most patients with CAVFs especially those which are first diagnosed before 20 years old are asymptomatic, and most symptoms and complications are detected after 20 years of age. On the other hand, the continuous murmur is not always detected in children or infants, consequently the cases of spontaneous closure may remain undetected. 

All symptomatic patients and those asymptomatic ones with moderate to severe shunting should be operated and minimal morbidity and good surgical results could be expected .However, because progression is unavoidable, medical managements are necessary, and the time of intervention is the primary focus in asymptomatic patients with no or only mild shunting.

## Notes

**Funding**: This study was supported by Iran University of Medical Sciences.

## References

[1] Parra-Bravo JR, Beirana-Palencia LG (2003). Right coronary artery fistula opening into right ventricle. Echocardiographic findings and interventional management. Report of a case. Arch Cardiol Mex.

[2] Blaschke F, Baur A, Roser M (2012). Absent proximal right coronary artery with a fistula into the pulmonary vein. Europace.

[3] Gupta A, Saxena S (2012). Acquired (post-angioplasty) coronary ventricular fistula draining into left ventricle aneurysm. J Invasive Cardiol.

[4] Luo L, Kebede S, Wu S, Stouffer GA (2006). Coronary artery fistulae. Am J Med Sci.

[5] Dresios C, Apostolakis S, Tzortzis S, Lazaridis K, Gardikiotis A (2010). Apical hypertrophic cardiomyopathy associated with multiple coronary artery-left ventricular fistulae: a report of a case and review of the literature. Eur J Echocardiogr.

[6] Gowda RM, Vasavada BC, Khan IA (2006). Coronary artery fistulas: clinical and therapeutic considerations. Int J Cardiol.

[7] Lowe JE, Oldham HN, Sabiston DC Jr (1981). Surgical management of congenital coronary artery fistulas. Ann Surg.

[8] Levin DC, Fellows KE, Abrams HL (1978). Hemodynamically significant primary anomalies of the coronary arteries. Angiographic aspects. Circulation.

[9] Tirilomis T, Aleksi L, Busch T (2005). Congenital coronary artery fistula in adult : surgical treatment and outcome. Int J Cardiol.

[10] Ata Y, Turk T, Bicer M (2009). Coronary arteriovenous fistulas in the adults: natural history and management strategies. J Cardiothorac Surg.

[11] Kouchoukos NT, Blackstone EH, Hanley FL, Kirklin JK ( 2012). Kirklin/Barratt-Boyes cardiac surgery.

[12] Chen JP, Rodie J (2009). Bi-directional flow in coronary-to-left ventricular fistula. Int J Cardiol.

[13] Mangukia CV (2012). Coronary artery fistula. Ann Thorac Surg.

[14] Sapin P, Frantz E, Jain A, Nichols TC, Dehmer GJ (1990). Coronary artery fistula: an abnormality affecting all age groups. Medicine (Baltimore).

[15] Reagan K, Boxt LM, Katz J (1994). Introduction to coronary arteriography. Radiol Clin North Am.

[16] Raju MG, Goyal SK, Punnam SR (2009). Coronary artery fistula: a case series with review of the literature. J Cardiol.

[17] Sherwood MC, Rockenmacher S, Colan SD, Geva T (1999). Prognostic significance of clinically silent coronary artery fistulas. Am J Cardiol.

[18] Angelini P, Angelini P, Fairchild VD (1999). Normal and anomalous coronary arteries in humans. Coronary artery anomalies: a comprehensive approach. In: Coronary artery anomalies. Illustrated edition.

[19] Carrel T, Tkebuchava T, Jenni R, Arbenz U, Turina M (1996). Congenital coronary fistulas in children and adults: diagnosis, surgical technique and results. Cardiology.

[20] Liberthson RR, Sagar K, Berkoben JP, Weintraub RM, Levine FH (1979). Congenital coronary arteriovenous fistula. Report of 13 patients, review of the literature and delineation of management.. Circulation.

[21] Eidem BW, Cetta F, O'Leary PW, Frommelt MA, Frommelt PC (2012). Echocardiography in pediatric and adult congenital heart disease. Vascular abnormalities.

[22] Ismail AQ, Gandhi A, Desai T, Stumper O (2012). A neonatal case of congenital coronary artery fistula. BMJ Case Rep.

[23] Farooki ZQ, Nowlen T, Hakimi M, Pinsky WW (1993). Congenital coronary artery fistulae: a review of 18 cases with special emphasis on spontaneous closure. Pediatr Cardiol.

[24] Schleich JM, Rey C, Gewilling M, Bozio A (2001). Spontaneus closure of congenital coronary artery fistula. Heart.

[25] Said SA, Nijhuis RL, Op Den Akker JW (2011). Diagnostic and therapeutic approach of congenital solitary coronary artery fistulas in adults: Dutch case series and review of literature. Neth Heart J.

[26] Raju MG, Goyal SK, Punnam SR (2009). Coronary artery fistula: a case series with review of the literature. J Cardiol.

[27] Lowe JE, Sabiston DC Jr, Sabiston DC Jr, Spencer FC (1983). Congenital malformations of the coronary circulation. Gibbon’s surgery of the chest.

[28] Balanescu S, Sangiorgi G, Castelvecchio S, Medda M, Inglese L (2001). Coronary artery fistulas: clinical consequences and methods of closure: a literature review. Ital Heart J.

